# A hypothesis for temporal coding of young and mature granule cells

**DOI:** 10.3389/fnins.2013.00075

**Published:** 2013-05-14

**Authors:** Lara M. Rangel, Laleh K. Quinn, Andrea A. Chiba, Fred H. Gage, James B. Aimone

**Affiliations:** ^1^Department of Neurosciences, University of CaliforniaSan Diego, CA, USA; ^2^Department of Mathematics and Statistics, Boston UniversityBoston, MA, USA; ^3^Department of Cognitive Science, University of CaliforniaSan Diego, CA, USA; ^4^Laboratory of Genetics, Salk Institute for Biological StudiesLa Jolla, CA, USA; ^5^Cognitive Modeling Department, Sandia National LaboratoriesAlbuquerque, NM, USA

**Keywords:** adult neurogenesis, oscillations, dentate gyrus, hippocampus, temporal coding, granule cells

## Abstract

While it has been hypothesized that adult neurogenesis (NG) plays a role in the encoding of temporal information at long time-scales, the temporal relationship of immature cells to the highly rhythmic network activity of the hippocampus has been largely unexplored. Here, we present a theory for how the activity of immature adult-born granule cells relates to hippocampal oscillations. Our hypothesis is that theta rhythmic (5–10 Hz) excitatory and inhibitory inputs into the hippocampus could differentially affect young and mature granule cells due to differences in intrinsic physiology and synaptic inhibition between the two cell populations. Consequently, immature cell activity may occur at broader ranges of theta phase than the activity of their mature counterparts. We describe how this differential influence on young and mature granule cells could separate the activity of differently aged neurons in a temporal coding regime. Notably, this process could have considerable implications on how the downstream CA3 region interprets the information conveyed by young and mature granule cells. To begin to investigate the phasic behavior of granule cells, we analyzed *in vivo* recordings of the rat dentate gyrus (DG), observing that the temporal behavior of granule cells with respect to the theta rhythm is different between rats with normal and impaired levels of NG. Specifically, in control animals, granule cells exhibit both strong and weak coupling to the phase of the theta rhythm. In contrast, the distribution of phase relationships in NG-impaired rats is shifted such that they are significantly stronger. These preliminary data support our hypothesis that immature neurons could distinctly affect the temporal dynamics of hippocampal encoding.

## Introduction

One of the most pronounced features of *in vivo* recordings within the hippocampus of rodents is the presence of robust theta frequency (5–10 Hz) oscillatory activity. The theta rhythm has garnered considerable attention for a number of reasons, mainly due to its strong correlates with behavior during a diverse array of tasks that suggest a functional role for this rhythm in learning, movement, attention, and sleep (Skaggs et al., 1996; Buzsáki, 2002). Importantly, both interneurons and principal cells of the hippocampus, including the granule cells of the dentate gyrus (DG), exhibit a strong relationship between the timing of their spiking activity and the phase of the underlying theta oscillatory activity of the network (Kubie et al., 1990; O'Keefe and Recce, 1993; Skaggs et al., 1996). It has been hypothesized that this precise timing serves to coordinate inputs in the hippocampus to be optimally read or ignored by downstream targets (Mizuseki et al., 2009; Buzsáki, 2010), and thus the tight or loose coupling of a cell's spiking activity to the underlying theta rhythm could have a potentially dramatic impact upon its influence within the network An unexplored area with regard to adult neurogenesis (NG) is the extent to which strong phasic activity in the hippocampal circuit may affect or be influenced by immature neurons during the course of their development.

There are many hypotheses regarding the source of the theta rhythm in the hippocampus and the mechanisms by which these sources may interact to influence or be driven by single cell activity. Specifically, several studies have suggested a local generation of the theta rhythm (Lee et al., 1994; Kocsis et al., 1999), septal generation (Mizumori et al., 1989; King et al., 1998; Yoder and Pang, 2005), as well as minor contributions from additional brain structures such as the supramammilary bodies (Ruan et al., 2010). This strong phasic input impacts the firing properties of DG granule cells in several ways. First, the principal excitatory input from the entorhinal cortex (EC) into the DG is largely theta rhythmic during exploratory behaviors, which comprise the most heavily studied behavioral condition from *in vivo* recordings of awake-behaving rats. These excitatory theta rhythmic projections from EC likely interact with the similarly rhythmic activity of local interneurons to gate input into the DG in a highly phasic manner. Additionally, as theta amplitude is modulated by septohippocampal projections, large levels of acetylcholine release are observed from medial septum (MS) and diagonal band of Broca (DBB) in the hippocampus during behaviors that evoke large increases in theta power, including REM sleep. These oscillatory inputs thus provide a potentially powerful mechanism by which cholinergic modulatory signals could influence how the DG and the rest of the hippocampus respond to cortical inputs (Bassant et al., 1995; Hasselmo, 1999; Heys et al., 2012).

There are several reasons to believe that immature neurons may have different temporal response properties to theta rhythmic input than mature neurons. Foremost is the fact that young neurons, particularly those less than a 6–8 weeks old, receive less synaptic inhibition than mature cells (Piatti et al., 2006; Li et al., 2012; Marín-Burgin et al., 2012). As a result, immature granule cells may not be subject to the same gating mechanisms imposed upon the network by the phasic activity of excitatory and local inhibitory inputs. Potentially just as impactful upon their participation in phasic dynamics of the network is the age-dependent physiology of granule cells. Young granule cells are known to respond differently to excitatory and inhibitory inputs, both in terms of their enhanced response to direct stimulation and in their increased ability to demonstrate plasticity during their development (Piatti et al., 2006; Toni et al., 2007). Furthermore, there is a simple statistical argument suggesting that young neurons, by virtue of their lower synaptic connectivity and increased relative excitability, are more likely to be driven by noise compared to mature neurons and, as a result, be potentially more responsive to novel inputs (Li et al., 2012). One consequence of this “small sample size” noise of young neurons is that *when* young neurons fire could be similarly affected by random fluctuations of their inputs. Overall these physiological characteristics suggest that immature granule cells are more broadly tuned than mature granule cells in response to specific inputs and in their relationships to oscillatory activity.

The temporal dynamics of immature granule cell participation in the theta rhythm are to date unknown. While several studies have used activity markers such as immediate early genes (IEGs) to identify active DG neurons, these methods are temporally coarse with resolution on the scale of minutes (Tashiro et al., 2007; Alme et al., 2010). Likewise, to understand the effects of the gradual integration of immature cells into the hippocampal network over weeks, computational models examining the differential responses of mature and immature granule cells over time typically study these effects over minute and day time-scales (Aimone et al., 2009; Aimone and Gage, 2011). Although it is possible using *in vivo* studies in the awake-behaving animal to resolve the temporal relationships between cell spiking behavior and theta oscillatory activity, the sparse firing properties of granule cells combined with the difficulty of identifying cell type using this technique has rendered it impossible to determine differences in these temporal relationships between immature and mature cells. As a result, multiple limitations have precluded insight into the finer temporal structure of granule cell activity.

A differential temporal response with respect to theta oscillatory activity between immature and mature neurons could have considerable implications upon memory encoding. The aforementioned tight temporal response of principal cells in the hippocampus with respect to theta suggests that it is not simply sufficient *that* an upstream DG neuron fires, but *when* it fires to its downstream targets in CA3 is important. Given that granule cells appear to fire fairly rarely *in vivo* (Leutgeb et al., 2007; Alme et al., 2010), their timing would appear to be critical, otherwise their effect would be minimized. Understanding how new neuron activity relates to the temporal structure of hippocampal activity is not only important from a functional point of view but also from an experimental point of view. As mentioned, one of the most substantial challenges in measuring the *in vivo* behavior of differently aged dentate granule cells is the absence of a clear physiological marker for young neurons. A clear temporal characteristic of immature granule cells would be of substantial value in estimating the age of recorded DG neurons. This paper will outline, in detail, the differences in the responses of immature and mature granule cells to their inputs, which could create unique temporal signatures with respect to the theta rhythm between the two cell types. Our hypothesis is based on two key observations: the inputs to GCs exhibit temporal behavior, and differently aged GCs respond differently to inputs. We explore each of these observations in detail below.

## Hypothesis and predictions

### Foundation no. 1: sets of input neurons have a temporal probability of being active at the same time, and these temporal probabilities are different for different combinations of neurons

**Figure 1:**
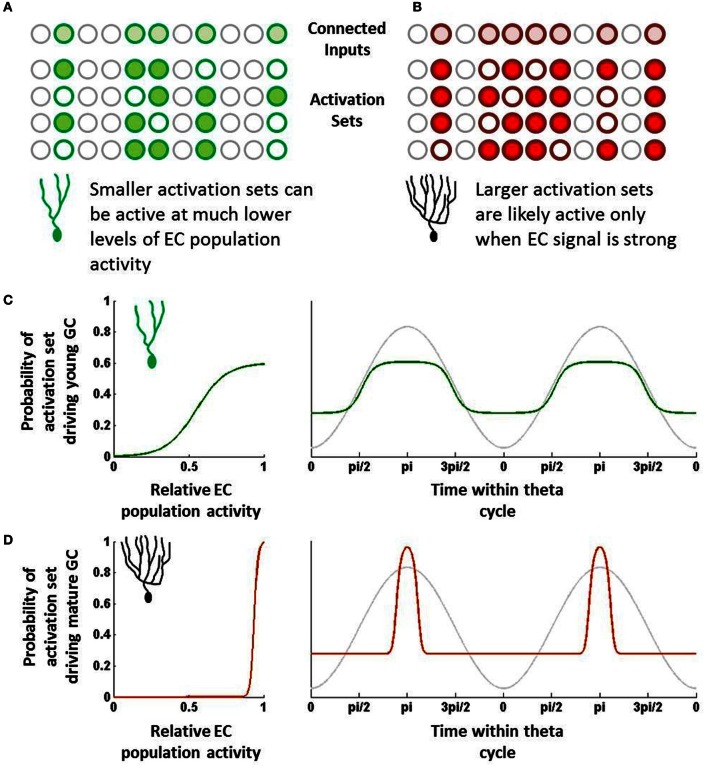
**Schematic overview of hypothesis. (A)** Young neurons have fewer inputs but can be driven by relatively few active inputs. These combinations of inputs, or “activation sets,” can be quite distinct from one another. **(B)** Mature neurons have many more input synapses but require many more inputs to be co-active to drive the neuron to fire. These larger activation sets would overlap one another more than those of young neurons. **(C)** Due to their small size, young neuron activation sets will be sufficiently active even at times when EC population activity is fairly low (left). As a result, if EC population activity is rising and falling with theta, young neurons would be capable of being activated at many different phases (green; right). **(D)** Due to their large size, mature neuron activation sets will likely only be active when EC activity is strongest and when that activity maps to what the mature cell codes for (left). This will result in only a relatively narrow band of theta that is sufficient to drive the mature neuron (red; right).

In cortical and hippocampal regions, neurons typically do not respond to single neurons but rather to sets of neurons that are active within a certain time window. Here, we will introduce the concept of an “activation set” for a neuron, which essentially can be defined as a group of neurons firing within a time range that is sufficient to drive a downstream neuron. Neurons do not require all of their synaptic inputs to be simultaneously active to fire, but rather sub-combinations of those are sufficient if coactive within a short amount of time. Individual neurons can thus have many different activation sets, the sizes of which may vary due to different synaptic weights (Figures 1A,B).

One can somewhat approximate the regulation of EC neurons by theta and other oscillations by assigning some probability of responding at any given time within a theta cycle. For instance, suppose an EC neuron's probability of firing can be represented by a sine wave; it fires rarely during troughs of theta and has a max firing rate at the peak (Deshmukh et al., 2010). This of course is oversimplifying the response, as oscillatory activity is far from the only indicator of when a neuron will fire. Still, over long periods of time and many different environments, theta is likely to be representative of the temporal dynamics of the EC population.

Other neurons in the EC population (as well as other neurons that project to granule cells, such as mossy cells) will have their own temporal tuning curves, which can vary in terms of amplitude (i.e., firing rate), preferred phase, and frequency (Deshmukh et al., 2010). Regardless of their shapes, combining the probabilities has the effect of multiplying sine waves, which provides increasingly narrower ranges of time in which the combined set may be active. Notably, some of these probabilities will be correlated with one another (neurons that tend to fire together), which will reduce this effect somewhat. However, as a first approximation, the probability that the set will be activated in full is the product of the individual neurons' temporal probability distributions.

Importantly, the shape and temporal position of this combined probability distribution depends on how many input neurons are parts of the set. The more neurons that contribute probabilities, the more narrow the combined temporal distribution becomes and the less likely the activation set is to be activated. Furthermore, the more neurons that compose the set, the closer the set's preferred phase will be to the overall population's preferred phase.

### Foundation no. 2: granule cells of different ages will have different sizes of preferred “activation sets” of input neurons

There is considerable evidence that young neurons are more responsive to inputs than mature neurons in spite of having considerably fewer synaptic inputs (Mongiat et al., 2009; Li et al., 2012; Marín-Burgin et al., 2012). Physiologically, it appears that the intrinsic properties of young neurons essentially compensate for fewer overall inputs, which has the effect of making individual synapses proportionately much stronger in young neurons. Previously, we have described how this difference could increase the statistical likelihood that young neurons could respond to novel inputs by chance as opposed to mature neurons (Li et al., 2012). By similar reasoning, with respect to the temporal responses, we can argue that the increased impact of individual synapses means that the set of inputs required to activate it can be fairly small (Figure 1A).

In contrast, mature neurons are typically highly inhibited and require considerable synaptic input to drive their activity. With regard to the temporal perspective described above, we would expect that most of the activation sets for a given mature neuron would require many input neurons to be coincidentally active, simply because individual synapses are proportionately much weaker on mature GCs. While the activation sets are not the same across different mature GCs, their basic properties with regard to theta (or other rhythms) would all be expected to be temporally very tight, essentially centered at the population average (say the peak of theta) (Figure 1B).

Importantly, however, we would also expect that mature GCs have some unique activation sets due to their past experiences. Learning has the effect of making important synapses more efficacious; in effect, this is the equivalent of making activation sets that include trained synapses that much smaller. So, like young neurons, these smaller activation sets may have off-the-mean phases and broader tuning. However, unlike immature neurons, these activation sets are not diverse, consisting primarily of those input sets comprising trained neurons.

### Main hypothesis

Mature neurons will have a strong preferred phase due to a response bias toward only the inputs to which they have been trained, whereas young neurons will demonstrate a broader phase relationship due to their responses to a wide range of different inputs.

As an approximation, the temporal firing probability distribution of a GC can be determined by summing all of that neuron's activation sets. Given the large numbers in the EC-DG circuit and relatively low firing rates, the probability that any single activation set drives behavior is quite low; however, the summation of many sets will be representative of the target neuron's temporal activity profile.

The summation of input probability distributions for young neurons, which as described above have a fairly diverse range of activation sets, will tend to be fairly broad (Figure 1C). This is due to the observation that, if many different combinations of EC neurons can drive young neuron activity, the GCs can potentially get activated at almost any phase during which at least some EC neurons are active. Young neurons have many different activation sets, and since these sets are relatively small, their preferred phases can be quite distinct from each other. As a result, the young neurons can be activated over a fairly broad range of theta, making them fairly incoherent to the EC population.

In contrast, most combinations of EC neurons do not activate mature neurons; rather their behavior has been hypothesized to be driven by the specific co-activation of those neurons to which they have learned to be highly responsive. While individually those input neurons may have broad responses, the combination of their temporal probability distributions would be fairly tight, perhaps only occurring when the EC activity is strongest (Figure 1D). Outside of that temporal range, the mature GC is simply very unlikely to receive a combination of mostly untrained inputs that is sufficient to drive it to fire. As a result, the mature GC's tuning curve would be quite tight, particularly in comparison to immature neurons.

### Predictions

The above hypothesis is clearly condensed and abstracted from the realities of the biological system. Fortunately, predictions derived from this hypothesis can be examined in physiological recordings from the behaving rat. Our hypothesis makes two novel predictions that are, in principle, testable. (1) Individually, young neurons will be *phase incoherent* with reference to theta, whereas mature neurons will be *phase coherent*. (2) Mature neurons as a population will be coupled to a specific phase of theta, namely the point at which EC input is the strongest (the peak or trough depending on polarity). Of course, the young neurons, due to their individual incoherence, will not be coupled to one another or to the peak of theta.

Directly testing these predictions will require the development of new technological approaches to recoding neurons within the DG. The first challenge is the identification of young neurons within an *in vivo* recording, which is not currently feasible in awake-behaving rats. However, until a method such as optogenetic identification is fully developed, we can use NG knockdown studies as an indirect surrogate. The second challenge is more subtle. Testing the second prediction—that there is a population phase coherence among mature neurons—not only requires the age identity of neurons but also a sufficiently large population recorded with a common oscillation reference. Current DG recording techniques typically can isolate at most only a few neurons simultaneously; we can only accumulate significant numbers of GCs by combining across tetrodes and animals for which the reference theta is different.

Accepting these limitations, we have analyzed data from a separate *in vivo* study of DG function in which NG has been transiently reduced in one cohort of animals (Rangel et al., under review). Within this study, we have recorded from numerous DG neurons (putative GCs) in both control rats and rats administered an anti-cell proliferation drug, temozolomide, which is known to reliably reduce adult NG in rodents (Garthe et al., 2009; Nokia et al., 2012). The study involved rats experiencing a set of distinct contexts at different times; however, we do not expect that these training differences will affect the phasic response of the neurons that we are examining. Here, we specifically have looked at the activity of different GCs during periods in which theta oscillations were strong, noting their phase coherence and preferred phase. Our prediction, based on the hypotheses above, is that granule cells in NG knockdown rats will exhibit stronger phase coherence than granule cells in control rats with intact NG.

## Methods

### Rats

All animal procedures were performed in accordance with NIH and local IACUC guidelines. Twelve adult, male, 300–350 g Long-Evans rats (Harlan Laboratories) were used as subjects. The rats were housed individually and maintained on a 12-h light/dark cycle, with all behavioral testing performed during the rats' light cycle. They were acclimated to the colony room for 3 days and handled daily for at least 2 weeks prior to beginning the experiment. Rats were given unrestricted access to water, but were placed on food restriction until they reached 85–90% of *ad libitum* weight to encourage foraging for food during the experiment. At the time of surgery, rats were approximately 3 months old at the time of surgery.

### Microdrive implantation surgery

**Figure 2:**
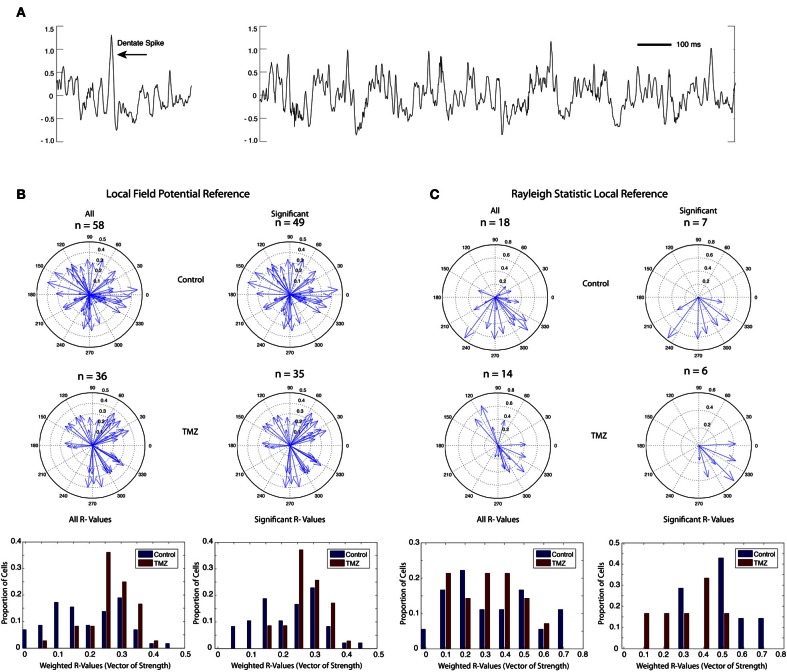
**Phase coherence of saline-treated and TMZ-treated rats to DG LFPs. (A)** Sample LFP trace (mV) showing typical dentate spike (left) and theta rhythm (right) in DG. **(B)** Phase coherence calculated by weighting the contribution of each spike by the power of theta at the time of the spike. Upper: vectors of strength (magnitude) and preferred phase angles summarizing the phase coherence of all identified granule cells in saline-treated rats (left) and granule cells with significant phase relationships to local theta rhythm (right). Middle: vectors of strength and preferred phase angles of all identified granule cells in TMZ-treated rats (left) and granule cells with significant phase relationships to local theta rhythm (right). Lower: histograms summarizing the distribution of vectors of strength (phase coherence magnitude) in all cells (left) and in cells with significant phase relationships only (right). Data are presented from both saline-treated (blue) and TMZ-treated (red) groups. **(C)** Same as **(B)** with phase coherence calculations restricted to spatially related firing during circular track running behavior only using a Rayleigh statistic.

Microdrives containing four tetrodes of 17 μm platinum iridium wire were surgically implanted using stereotaxic procedures (from bregma A/P: −4.0, M/L: +2.2 mm, D/V: −2.2 mm). Tetrode bundles were then advanced in 10 μm increments each day until they reached the granule cell layer (D/V: ~2.7 mm) as indicated by the appearance of place cell single units, “dentate spikes,” and complex high frequency (16–90 Hz) local field potential (LFP) activity (Figure 2A). Wires were not turned within 24 h prior to a recording session. A skull screw over frontal cortex served as a ground electrode, and a skull screw over cerebellum served as a reference for LFP activity.

### Neural recordings

At the start of each recording session, each wire was first checked for neural activity, with the quietest wire chosen to serve as a reference electrode for single unit activity. The activity on each wire was passed through a high-impedance OpAmp headstage that held a set of light-emitting diodes (Neuralynx Technologies, Bozeman, MT). A multiwire flexible cable connected the preamplifiers to a 32-channel commutator (Neuralynx Technologies, Bozeman, MT). The single-unit signal was bandpass filtered between 600 and 6000 Hz and amplified through Neuralynx Lynx-8 differential programmable amplifiers. The LFP signal was bandpass filtered between 1 and 475 Hz. The amplifiers were integrated with the Cheetah data acquisition program (Neuralynx Technologies, Bozeman, MT), wherein the acquired analog signals were digitally converted at a rate of 30,303.00 KHz (DT 2821 Data Translation, Marlboro, MA) prior to storage. The position of the rat was monitored by a set of light-emitting diodes placed on the headstage. A video tracking system (SA-2 Dragon Tracker, Boulder, CO) registered the position of the diodes with a sampling frequency of 30 Hz. All recording sessions began with 5 min of baseline recording activity in a home cage environment. Single cells were isolated using Offline Sorter (*Plexon*, Dallas, TX) by comparing relative amplitude and other parameters of each spike across tetrode wires. Final microdrive wire location was verified post-mortem in 40-um sections using a Nissl stain.

### Behavior

Rats were presented with three different behavioral contexts during each recording session: (1) a 13″ × 13″ box in which ¼ pieces of Honey Nut Cheerios were mixed with TEK-Fresh bedding (Harlan Laboratories) and placed randomly throughout the box; (2) a 48″ diameter black circular track with a 3″ wide pathway in which rats were rewarded with ¼ pieces of Honey Nut Cheerios in a reliably rewarded location for >15 laps that was shifted up to three times per session to a different track location; and (3) a 48″ diameter circular cheeseboard with chocolate sprinkles spaced randomly throughout the context and three presentations of a large food reward (a 2″ diameter dish filled with chocolate sprinkles) at random time intervals and random locations for a period of >30 s. All three behavioral contexts were presented in the same location of the same recording room surrounded by a black curtain to minimize external cues. All behavioral and recording sessions were performed with the room lights turned off. To allow full exploration of the larger contexts and minimize oversampling of the smaller contexts, the exposure lengths to the foraging pot, circular track, and circular cheeseboard were approximately 10, 15, and 20 min, respectively.

### Adult neurogenesis knockdown

Temozolomide (TMZ) is an anti-cell proliferation drug that has been shown to reduce cell proliferation in the DG by more than 80% in mice (Garthe et al., 2009). After testing several dosages of the drug in rats, we found the optimal dosage to be three intraperitoneal injections of 12.5 mg/kg each week. Notably, TMZ has a transient effect on NG. To quantify the time course of its efficacy in our experiment, we injected a separate group of rats with either TMZ or saline using this injection regime. We then additionally injected rats with BrdU (4 consecutive days; 50 mg/kg) at either 11 days (second week) or 25 days (fourth week) after TMZ onset to measure proliferation. We observed that after 2 weeks of TMZ treatment, there was a significant reduction in the number of BrdU-labeled cells with respect to saline-injected controls (65% reduction, mean of saline-treated = 1767 ± 333 and mean of TMZ-treated = 1144 ± 398 for total number of BrdU-labeled cells, two-tailed *t*-test, *df* = 5, *p* = 0.0065), whereas after 4 weeks of TMZ there was no significant difference between TMZ or saline-treated groups. These results are consistent with previous studies using a similar injection regime (Nokia et al., 2012). To prevent possible side effects from stopping treatment after the beginning of the experiment, three injections of 12.5 mg/kg TMZ were performed every week for 10 weeks. *In vivo* electrophysiological recordings were acquired 10 weeks after the first TMZ injection, thus capturing a NG knockdown of approximately 35% in the 6- to 10-week-old neuron populations by the time of the recording experiment.

### Analysis of theta phase relationships

To assess changes in the phase relationship of single unit spikes to the local theta rhythm, the phase of each spike for each cell was first determined from the filtered Hilbert transform (Butterworth filtered between 5 and 10 Hz). The contribution of each spike phase, θ, to the overall vector of strength *R* was determined using the following equation:

$$R=\sqrt{X_{w}^{2}+Y_{w}^{2}}$$

where

$$\begin{array}{l} X_{w}=\frac{\sum w\times \cos(\theta )}{\sum w}\\ Y_{w}=\frac{\sum w\times \sin(\theta )}{\sum w} \end{array}$$

and *w* is the power of theta at the time of a given spike divided by the maximum theta power over the course of a recording session. Statistical significance of *R* was defined as the 95% confidence interval in a distribution of one thousand random *R*-values generated from random spike times equal in number to those acquired from a given cell for each recording session.

A more traditional method was also used that calculated *R* using a Rayleigh statistic only from spikes acquired as a rat ran through a given cell's determined place field during running on a circular track. Only those cells exhibiting a place field of at least 5 Hz and a diameter greater than 10 cm were included in this second analysis. Place field boundaries were defined by the points at which firing rate was reduced to 10% of peak. Only cells with a mean firing rate for the entire recording session that was above 0.05 Hz and below 3 Hz were utilized to ensure sufficient data samples for analysis and exclusion of interneurons, respectively.

## Results

Rats in both saline-treated and TMZ-treated groups received single exposures to three different behavioral contexts during each recording session: a forage box in which they foraged for randomly spaced food reward, a circular track in which they ran in a circle for a food reward in a reliably rewarded location, and a circular cheeseboard arena in which they again foraged for a randomly spaced food reward and received three presentations of a large food reward at random times and at random locations. Thus, each of the three contexts required different behaviors. To quantify phase relationships of single cells to dentate LFPs across these different behaviors, we developed a new method of calculating phase relationships that weighted the contribution of each spike by the power of theta at the time of the spike (see Methods). This would allow stronger contributions from spikes at high instances of theta and weaker contributions from cells at low instances of theta. A total of 26 recording sessions were acquired from 7 rats in the saline-treated control group and 23 recording sessions from 6 rats in the TMZ-treated group. We found no significant difference in theta power across groups (two-tailed *t*-test *p* = 0.69, *df* = 47).

Fifty-eight granule cells recorded from 7 rats in the saline-treated group and 36 granule cells from 6 rats in the TMZ-treated group had to meet a minimum firing rate criterion of 0.05 Hz on the circular track context to be included for phase analysis. Cells acquired from the TMZ-treated group demonstrated greater phase relationships to DG LFPs than cells acquired from the saline-treated group (Figure 2B, two-tailed *t*-test *p* = 0.0016, *df* = 92). A large majority of the phase relationships calculated for both groups were significant (Figure 2B, right panels), indicating that they demonstrated a phase relationship to theta above what would be expected by chance. Of the cells with significant phase relationships, those acquired from TMZ-treated rats still exhibited higher phase relationships than those acquired from saline-treated rats (two-tailed *t*-test *p* = 0.0183, *df* = 81).

Our novel method of calculating theta phase relationships captures spiking activity that is not necessarily related to spatial encoding and not restricted to behavioral epochs in which theta power is maximal. We additionally calculated a Rayleigh statistic on data restricted to place field activity during circular track behavior (see Methods). Using this method, we found a total of 18 cells in the saline-treated group and 14 cells in the TMZ-treated group that exhibited place fields on the circular track (Figure 2C). Of these cells, only 7 cells in the saline-treated group and 6 cells in the TMZ-treated group exhibited significant phase relationships to theta. There were no significant differences in the strength of phase relationships across groups using this measure (comparison between all cells: two-tailed *t*-test, *p* = 0.7325, *df* = 30, comparison between cells with significant phase coherence only: two-tailed *t*-test, *p* = 0.0575, *df* = 11).

## Discussion

As predicted, the TMZ group, which presumably has a higher percentage of mature neurons, showed significantly higher phase coherence to local theta, which is consistent with the hypothesis that immature neurons are more loosely tuned to theta. Notably, our experimental injection paradigm resulted in a partial knockdown of neurons that were approximately 6–10 weeks old. This finding suggests that a full knockdown of adult-born neurons spanning a wider age range would produce a more pronounced effect. While our unique measure of phase coherence revealed a significant difference in the strength of coherence between groups, a similar comparison using only spatial activity on the circular track context demonstrated no significant difference. This may in part be due to the sparse firing properties of the granule cell population such that a limited number of cells qualified as exhibiting spatial preference or place cell activity, thus reducing the number of cells available for comparison. Moreover, analyses restricted only to spatial activity may unequally sample activity from mature and immature neurons, as it is not known at what age dentate granule cells exhibit the spatially selective activity of typical place cells in the hippocampus. Thus, whereas our second analysis ensured that we only examined granule cell activity during behaviors that could elicit high theta power, this restriction may unnecessarily mask differences in the temporal firing properties of neurons from both groups by only considering spatially related activity.

**Figure 3:**
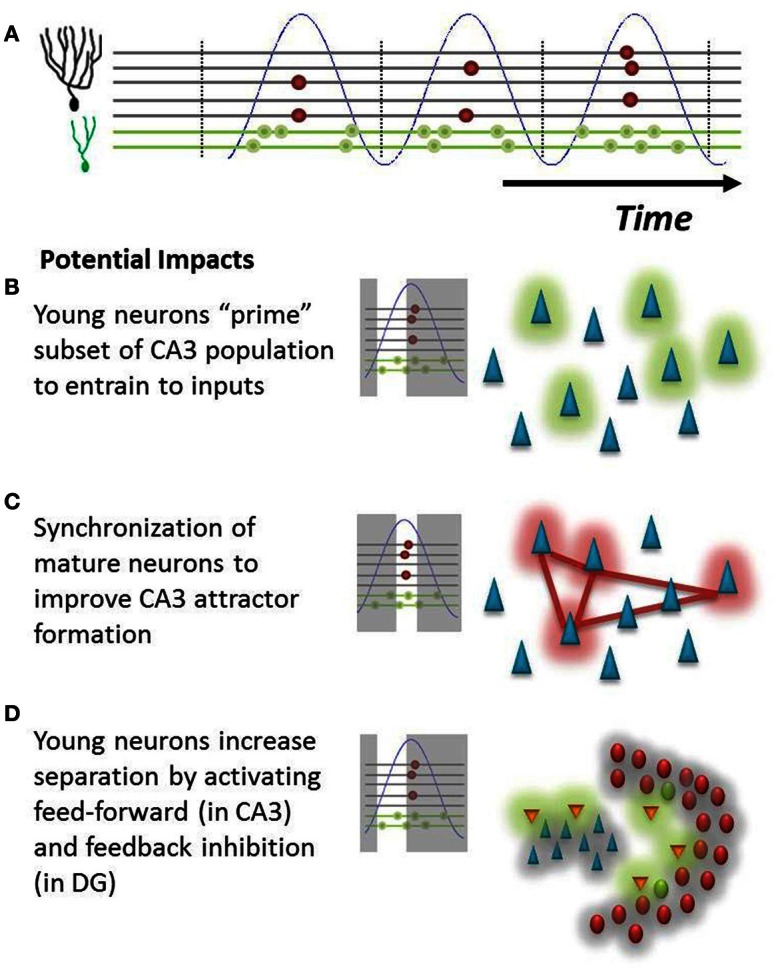
**Potential impact of hypothesis on DG-CA3 relationship. (A)** Illustration of population behavior predicted by hypothesis. Young neurons (green dots) will fire at many phases of theta (blue), whereas mature neurons (red dots) will fire at only the peak of EC population activity. **(B)** Early in the theta oscillation, young neurons will fire onto CA3 neurons, possibly priming a subset of pyramidal cells (green cloud) for the subsequent formation of new attractors. **(C)** Synchronized mature neurons could more effectively drive a population of interconnected pyramidal cells (red cloud) in CA3, driving memory attractor formation (red lines). **(D)** Young neurons could preferentially activate inhibitory networks (orange neurons) in both CA3 and in the hilus, subsequently inhibiting neurons throughout both regions (gray cloud) and potentially increasing network pattern separation.

Beyond the possible ability to identify immature neurons described above, a differential temporal response of young and mature neurons introduces interesting considerations from a functional perspective. Our hypothesis suggests that young neurons will fire at a much broader range of the theta oscillation. This provides new neurons with several non-exclusive mechanisms to influence hippocampal function. (1) An immature GC signal onto CA3 neurons could precede that of mature neurons by as much as several dozen milliseconds during a theta cycle, giving these potentially weaker inputs that presumably represent novel features a “head-start” to bias the CA3 population for later encoding (Toni et al., 2008; Aimone et al., 2011; Gu et al., 2012); (2) the increased phase coherence of mature neurons could allow them to reliably encode familiar features into novel attractors in the CA3 network; and (3) likewise, if immature neurons are the first population to be activated, they would be ideally suited to activate feed-forward inhibition in the CA3 (Lawrence and McBain, 2003), which could increase attractor separation (Aimone et al., 2010) as well as feedback inhibition pathways within the DG (Figure 3). This latter function is consistent with that proposed by Sahay et al. (2011). The potential impact of this feedback inhibition could be quite powerful in response to novel events for which other mature GCs have no learned response. While mature GCs may be weakly responsive to a novel event, one could imagine that they require a longer period of time than immature neurons to integrate non-optimal signals. If there are young neurons in the network that respond earlier, they could in effect keep mature neurons from responding to less than ideal (untrained) inputs. In this manner, the young neuron could learn to respond to the novel event while simultaneously sharpening the tuning curves of its mature “competitors.”

An alternative hypothesis is that the firing incoherence of immature cells actually reduces their impact upon downstream hilar and CA3 targets until they have reached a mature age, in effect minimizing the potentially disruptive impact of immature neuron behavior on the mature circuit. One would expect that the effects of inputs on CA3 and other downstream neurons would be highly phase dependent, just as we have described how DG responses might depend on phase. It may be that the phasic responses of CA3 neurons, which themselves are highly coupled to theta, make them less likely to register mossy fiber inputs received at non-optimal phases of theta. Thus, further evidence will be necessary to confirm that immature neurons have any influence upon their downstream CA3 targets prior to their full integration into the mature circuit.

These potential implications on the downstream CA3 circuit add to the growing uncertainty about how young neurons influence hippocampal function beyond the DG and suggest that any study that seeks to investigate these relationships *in vivo* should take the temporal structure of DG activity into account. Notably, the relatively early and late temporal responses of immature neurons with respect to their mature counterparts could additionally affect plasticity in CA3 that is spike timing dependent (STDP). This is also an added consideration for optogenetic studies, whose induced activity of granule cells is typically uncoupled to the intrinsic network dynamics (Gu et al., 2012). Furthermore, as mentioned above, the data presented here only provide limited support for our hypothesis. Complete validation will require a full characterization of how a much larger population of granule cells responds to oscillatory influences as well as a direct measure of neuronal age. In addition, it would be interesting to apply techniques for acutely reducing theta modulation of the EC in behavioral studies thought to involve new neurons (Brandon et al., 2011; Koenig et al., 2011).

In summary, the hypothesis and supporting data presented here suggest that the phasic temporal behavior of young and mature neurons is non-trivial and merits further consideration and attention in theoretical interpretations of both NG and DG function.

### Conflict of interest statement

The authors declare that the research was conducted in the absence of any commercial or financial relationships that could be construed as a potential conflict of interest.
